# ZAP-70 Promotes the Infiltration of Malignant B-Lymphocytes into the Bone Marrow by Enhancing Signaling and Migration after CXCR4 Stimulation

**DOI:** 10.1371/journal.pone.0081221

**Published:** 2013-12-03

**Authors:** Eva Calpe, Noelia Purroy, Cecilia Carpio, Pau Abrisqueta, Júlia Carabia, Carles Palacio, Josep Castellví, Marta Crespo, Francesc Bosch

**Affiliations:** 1 Laboratory of Experimental Hematology, Department of Hematology, Vall d'Hebron University Hospital, Universitat Autònoma de Barcelona, Barcelona, Spain; 2 Department of Pathology, Vall d'Hebron University Hospital, Universitat Autònoma de Barcelona, Barcelona, Spain; University of Manitoba, Canada

## Abstract

ZAP-70 in chronic lymphocytic leukemia (CLL) is associated with enhanced response to microenvironmental stimuli. We analyzed the functional consequences of ZAP-70 ectopic expression in malignant B-cells in a xenograft mouse model of disseminated B-cell leukemia. Mice injected with B-cells expressing ZAP-70 showed a prominently higher infiltration of the bone marrow. *In vitro* analysis of the response of malignant B-cells to CXCL12, the main attracting chemokine regulating trafficking of lymphocytes to the bone marrow, or to bone marrow stromal cells, revealed that ZAP-70 induces an increased response in terms of signaling and migration. These effects are probably mediated by direct participation of ZAP-70 in CXCL12-CXCR4 signaling since CXCR4 stimulation led to activation of ZAP-70 and downstream signaling pathways, such as MAPK and Akt, whereas ZAP-70 did not alter the expression of the CXCR4 receptor. In addition, subclones of primary CLL cells with high expression of ZAP-70 also showed increased migrative capacity toward CXCL12. Neutralization of CXCR4 with a monoclonal antibody resulted in impaired *in vitro* responses to CXCL12 and bone marrow stromal cells. We conclude that ZAP-70 enhances the migration of malignant B-cells into the supportive microenvironment found in the bone marrow mainly by enhancing signaling and migration after CXCR4 stimulation.

## Introduction

Chronic lymphocytic leukemia (CLL) cells found in the peripheral blood are mainly in the G_0_ phase of the cell cycle whereas CLL cells located in lymphoid organs and in the bone marrow find a favorable microenvironment. In these organs CLL cells receive survival, anti-apoptotic and proliferative signals, being the amount of actively proliferating cells directly related to prognosis [1], [2]. These stimuli are mainly mediated by cytokine receptors [3], [4], the B-cell receptor (BCR) [5] and other surface molecules such as CD40, Toll-like receptors and BAFF-R [6]–[8].

High expression of ZAP-70 protein is a strong predictor of higher probability of progression and shorter overall survival [9]–[11]. Despite recent advances, the complete picture of the role of ZAP-70 in the biology of B-cell malignancies is still not fully defined. One of the reasons for this is the confounding effect of many different factors associated with ZAP-70 expression in primary CLL cells. Notwithstanding, there is accumulating data about the role of ZAP-70 in the crosstalk between CLL cells and the microenvironment. Thus, ZAP-70 expression in CLL cells has been related to enhanced signaling through the BCR, and to increased response to diverse migrative and survival stimuli from the microenvironment [12]–[18]. As previously described for normal B-lymphocytes [19], [20]. stimulation of the BCR in CLL cells can lead to a modulation of the expression of different chemokine receptors and adhesion molecules [14], [21], [22], which can be influenced by the presence of ZAP-70 [14].

Against this background, we aimed to ascertain the specific influence of ZAP-70 protein in the infiltrative capacity of malignant B-lymphocytes by using an established xenograft mice model of disseminated B-cell leukemia. In this model, ZAP-70 was the only variable between groups. We found that ectopic expression of ZAP-70 increased the capacity of malignant B-cells to infiltrate the bone marrow via enhancement of the response to CXCR4 stimulation in terms of signaling and migration.

## Materials and Methods

### Ethics statement

Animal studies were performed in accordance with the institutional guidelines set by the Vall d'Hebron University Hospital Care and Use Committee (protocol approved under permit number 77/11). All mice were euthanized under anesthesia and experienced no pain or suffering. All patient samples were obtained following a protocol approved by the Clinical Research Ethics Committee (CREC) of the Vall d'Hebron University Hospital according to the principles of the Declaration of Helsinki after written informed consent.

### Cell lines and primary cells

The Burkitt's lymphoma B-cell line Raji and the Jurkat T-cell line were obtained from American Type Culture Collection (ATCC, Manassas, VA, USA). The murine bone marrow stromal cell (BMSC) cell line MS-5 was kindly provided by Dr. Barquinero (Laboratory of Gene and Cell Therapy, Vall d'Hebron Institut de Recerca, Barcelona, Spain) [23]. Cell lines were cultured in RPMI-1640 or DMEM medium (MS-5) supplemented with 10% heat-inactivated fetal bovine serum (FBS), 100 U/mL penicillin, 0.1 mg/mL streptomycin and 2 mM L-glutamine at 37°C in a 5% CO_2_ atmosphere. The GFP-ZAP-70 expression vector (pEGFP-N2ZAP-70) was generated as previously described.[16]. Raji cells were stably transfected with plasmids expressing either GFP-ZAP-70 fusion protein or GFP only as a control as previously described [16]. Briefly, cells were electroporated (150 µF/300 V) and subsequently selected for the presence of the plasmids in standard growth medium containing 1.2 mg/ml of G418 (Invitrogen), and further sorted by GFP expression. Mononuclear cells from peripheral blood from 50 patients with CLL were obtained by Ficoll-Paque Plus (GE healthcare, Buckinghamshire, England) density gradient.

### Disseminated B-cell leukemia xenograft model

Seven- to 9-weeks old female C.B-17 SCID mice were purchased from Charles River Laboratories (Barcelona, Spain). To establish the xenograft model, 2×10^6^ Raji GFP or Raji GFP-ZAP-70 cells in 200 µL of Phosphate Buffered Saline (PBS) solution were injected into the tail vein of mice. Mice were examined daily for general condition and hind leg paralysis. Animals were sacrificed when they developed hind leg paralysis, and all efforts were made to minimize suffering.

### Immunohistochemistry

Organs from euthanized mice were obtained, and specimens from bone marrow (femur), brain, spleen, lung, liver, kidney, submaxilar lymph node and spinal cord were rinsed with PBS, fixed with 4% formaldehyde and embedded into paraffin blocks. Sections (2-3 µm thick) were deparaffinized by incubation at 60°C for 1 hour followed by immersion in xylene and rehydrated through graded alcohol washes. The BenchMark® XT automated slide processing system (Ventana Medical Systems, Oro Valley, AZ, USA) was used for GFP detection using anti-GFP antibody (Santa Cruz Biotechnology, Shandon, CA, USA) at 1/500 dilution.

### Flow cytometry

The expression of cell surface antigens was detected using the following fluorochrome-labeled antibodies: CXCR4-allophycocyanin (APC), CD3-phycoerythrin-cyanine 7 (PE-C7) (BD Biosciences, San Jose, CA, USA), CD19-PE-Texas Red (ECD) and CD5-PE-cyanine 5.5 (PC5.5) (Beckman Coulter, Brea, CA, USA). For detection of intracellular ZAP-70 in primary CLL cells, IntraSure kit and primary antibody anti-ZAP-70-PE (Beckman Coulter) were used. GFP expression was analyzed in single cell suspensions from mice organs rinsed with PBS, minced and filtered through a cell strainer. Bone marrow cells were obtained by rinsing the femur cavity with PBS. Peripheral blood was collected from the vena cava of anesthetized mice. Cells were acquired in a Navios cytometer (Beckman Coulter) and results evaluated using FCS Express Version 4 software (De Novo Software, Los Angeles, CA, USA).

### Immunoblotting

Stimulation of CXCR4 with 100 ng/mL of CXCL12 (PeproTech, London, England) was performed in Raji cells for the indicated time points. Jurkat cells treated with the phosphatase-inhibitor pervanadate (3 mM H_2_O_2_/1 mM NaVO_4_) for 5 minutes at 37°C were used as positive controls for phospho-proteins. One million cells were pelleted and then lysed for 30 minutes at 4°C in 100 µL lysis buffer (20 mM Tris pH 7.4, 1 mM EDTA, 140 mM NaCl, 1% NP-40, 2 mM sodium vanadate and 1X proteases inhibitor cocktail; Sigma-Aldrich, St.Louis, MO, USA). Equal amounts of denatured protein were resolved by 10% SDS-PAGE and transferred to Immobilon-P membranes (Millipore, Bedford, MA, USA). Membranes were blocked for 1 hour at room temperature in 5% non-fat milk/TBST (Tris-Buffered Saline Tween-20) and incubated overnight at 4°C with primary antibodies (phospho-ZAP-70^Tyr319^/Syk^Tyr352^, phospho-Akt^Ser473^, phospho-ERK1/2^Thr202/Tyr204^, Akt and ERK1/2 (Cell Signaling Technology, Beverly, MA, USA), ZAP-70 (clone 2F3.2, Upstate Biotechnology, New York, NY, USA), and β-actin (Abcam, Cambridge, England) as loading control. Immunodetection was done using the ECL chemiluminescence detection system (GE Healthcare) and images were acquired with the LAS-4000 system (Fujifilm Life Science, Carrollton, TX, USA). Quantification of band density was performed using ImageJ software (Wayne Rasband, Bethesda, MA).

### Chemotaxis assay

Migration to the chemokine CXCL12 and to the murine BMSC cell line MS-5 was determined in Raji cells by using a transwell migration assay across bare polycarbonate membranes (Corning, New York, NY, USA). A total of 100 µL of RPMI-10% FBS containing 1×10^6^ cells was added to the top chamber of a 6.5-mm-diameter transwell culture insert with a pore size of 5 µm. For migration toward CXCL12, 600 µL of RPMI-10% FBS alone or with 100 ng/mL of CXCL12 were added to the lower chamber. For migration to BMSCs, 1.5×10^5^ MS-5 cells seeded and cultured overnight on DMEM-10% FBS or DMEM-10% FBS alone was added to the lower chamber. Chambers were incubated for 4 hours at 37°C in 5% CO_2_ and cells in the lower chamber were counted with a Navios cytometer under a defined flow rate for 5 minutes. The migration index was calculated as the number of cells transmigrating with chemokine or stromal cells divided by the number of transmigrating cells with control medium only. Chemotaxis assay of primary CLL cells from 10 patients was performed by adding 1 mL of RPMI-0.5% BSA containing 1.5×10^7^ cells to the top chamber of a 24-mm-diameter transwell culture insert. Cells were allowed to migrate toward 2.4 mL of media containing 200 ng/mL CXCL12 for 6 hours, and the percentage of CLL cells expressing ZAP-70 was then determined in both the upper and lower chambers by flow cytometry. For CXCR4 blocking, cells were treated with the monoclonal antibody anti-CXCR4 MAB171 (R&D Systems, Minneapolis, MN, USA) or the IgG2a isotypic control (BD Biosciences).

### Cell proliferation assay

Cell proliferation was measured using the Cell-Titer 96^TM^ Cell Proliferation Assay (Promega, Madison, WI, USA), which uses cellular conversion of a tetrazolium salt into a blue formazan product (MTS-assay). A total of 2.5×10^4^ Raji cells were seeded per well in a 96-well plate in 100 µL of RPMI-10% FBS alone or with 100 ng/mL of CXCL12. Plates were incubated for 24, 48 and 72 hours and absorbance was measured in a plate reader at 490 nm after MTS conversion.

### Cell viability

Cell viability was determined by flow cytometry using Annexin V-APC-Propidium Iodide (PI) staining (Bender MedSystems, Vienna, Austria) following the manufacturer's instructions. Cells were acquired in a Navios cytometer (Beckman Coulter) and results evaluated using FCS Express Version 4 software (De Novo Software).

### Statistical analysis

Results are shown as mean ± standard error of the mean (SEM) of at least 4 replicates or independent experiments. For statistical comparison between groups, the Mann-Whitney test or the non parametric paired-sample test (Wilcoxon) was used, and *P*<.05 was considered significant. Survival curves were generated using the Kaplan and Meier method, and analyzed by the log-rank test. Analyses were performed using the biostatistics software package SPSS Version 17 (IBM, Somers, NY, USA). Results were graphed with GraphPad Prism Version 5.0 (La Jolla, CA, USA).

## Results

### ZAP-70 expression greatly enhances the capacity of malignant B-cells to infiltrate the bone marrow in a xenograft model

**Figure 1:**
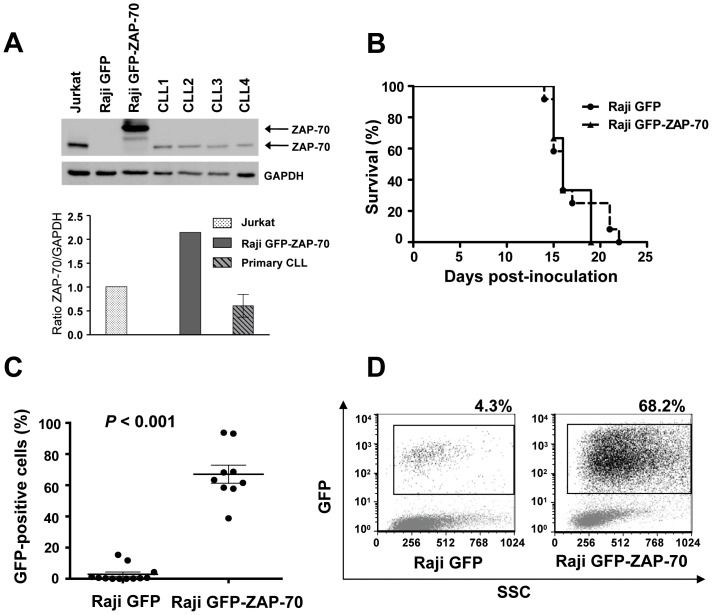
ZAP-70 ectopic expression enhances the capacity of malignant B-cells to infiltrate the bone marrow. SCID mice were intravenously injected with 2×10^6^ stably transfected Raji cells (12 mice with control Raji GFP cells and 9 mice with Raji GFP-ZAP-70 cells). (A) ZAP-70 expression levels were compared by western blotting in Raji transfectants and primary CLL samples. (B) Survival curves according to Kaplan and Meier method. (C) Percentage of GFP-positive cells as assessed by flow cytometry in the bone marrow from mice injected with Raji GFP-ZAP-70 cells (n = 9) and Raji GFP cells (n = 12). Graph shows the mean percentage ± SEM (D) Flow cytometry dot plot showing percentage of GFP-positive cells in the bone marrow from two representative mice.

Ectopic expression of ZAP-70 protein in malignant B-cells enhances BCR signaling and increases the expression and signaling of the chemokine receptor CCR7, which translates into an enhanced *in vitro* migration toward the CCL21 chemokine, as we showed in a previous study [16]. To further elucidate the role of ZAP-70 in the interactions between malignant B-cells and the microenvironment, Raji B-cells stably transfected with a vector expressing a GFP-ZAP-70 fusion protein or GFP only as a control, were intravenously injected into SCID mice. This widely described xenograft mouse model of disseminated B-cell leukemia develops hind legs paralysis at around 14 to 19 days after cell injection due to central nervous system infiltration [24], [25]. Paralysis precedes death by 1 to 2 days; therefore this event was considered the end point of the study. The expression level of ZAP-70 protein in the cell line was compared to that of a selection of samples from patients with CLL and high expression of ZAP-70 (n = 4) in order to discard possible effects due to supra-physiologic levels of ZAP-70 protein. As can be observed in Figure 1A, levels of ZAP-70 were around three fold higher compared to primary CLL cells.

**Figure 2:**
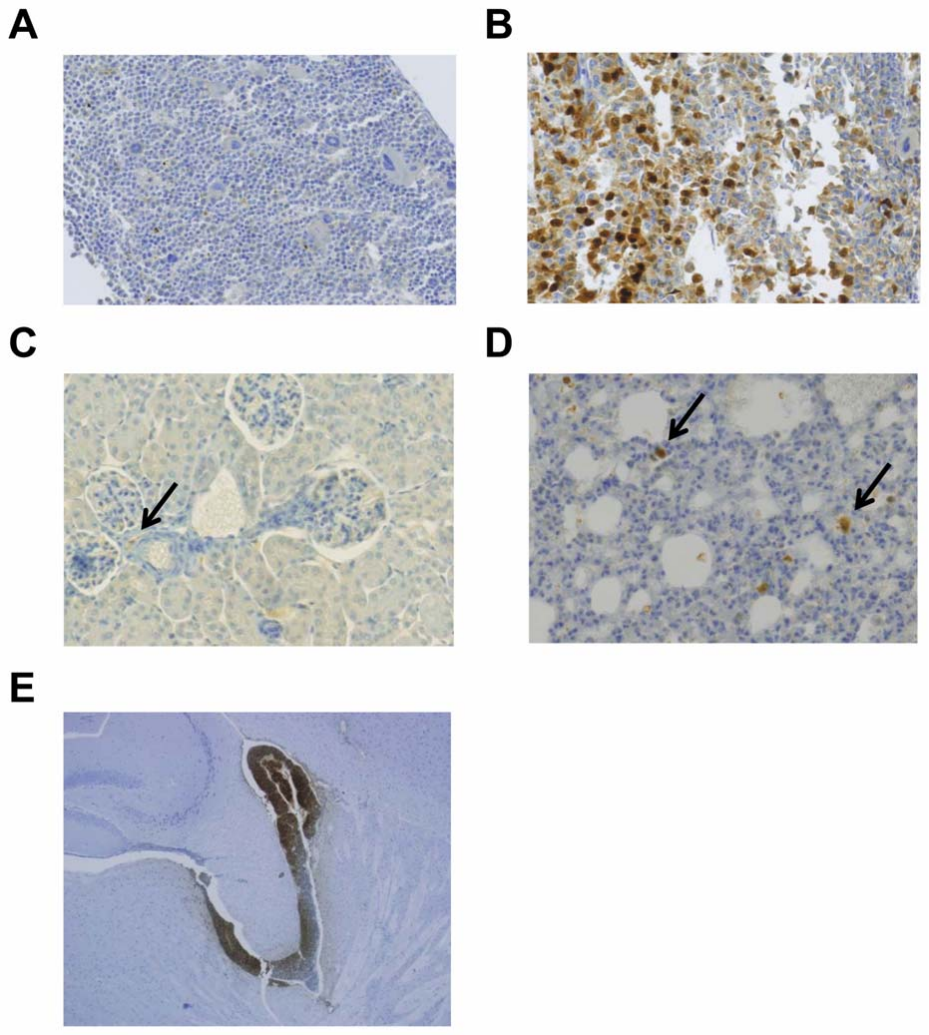
GFP expression in bone marrow, kidney, lung and brain from mice injected with Raji B-cells. GFP expression (*brown*) was analyzed by immunohistochemistry in fixed specimens of bone marrow Raji GFP (A), bone marrow Raji GFP-ZAP-70 (B), kidney (C), lung (D) (x20) and brain (E) (x4.2) from mice injected with Raji B-cells. Images from bone marrow show a representative mouse from each group and representative specimens from mice injected with Raji-GFP-ZAP-70 cells are shown in the other images. Slides were counterstained with hematoxylin.

Twelve mice were inoculated with Raji GFP cells and 9 mice with Raji GFP-ZAP-70 cells. Median survival for both groups of mice was 16 days (Figure 1B), indicating that the expression of ZAP-70 does not affect mice survival. In order to assess the influence of ZAP-70 in the infiltrative capacity of B lymphocytes, we analyzed the presence of malignant B-cells in different organs at the time of paralysis by assessing the amount of infiltrating GFP-positive cells by flow cytometry and immunohistochemistry in all animals. Strikingly, a higher infiltration of malignant B-cells was observed in the bone marrow of mice injected with Raji GFP-ZAP-70 cells compared to the bone marrow of mice inoculated with control Raji B-cells, as measured by flow cytometry (67%±5.76% vs. 2.9%±1.49%; *P*<.001; Figures 1C and 1D). This was corroborated by immunohistochemical analysis, which further revealed the higher infiltration of GFP-positive cells in the bone marrow from mice injected with Raji GFP-ZAP-70 cells (Figures 2A and 2B).

The percentage of B-cells infiltrating the additional organs analyzed was inferior to the one found in the bone marrow and, for most of the organs analyzed, it was significantly higher in mice injected with Raji GFP-ZAP-70 cells than in mice injected with Raji-GFP cells (Table 1). In agreement, scattered GFP-positive cells in both groups were detected by immunohistochemistry in the additional organs analyzed (Figures 2C and 2D). Of note, however, the analysis of spinal cord samples showed a massive meningeal infiltration in both groups of animals (Figure 2E); this similar pattern of meningeal infiltration by malignant-B lymphocytes regardless of their expression of ZAP-70, probably accounts for the simultaneous onset of the hind legs paralysis and therefore similar survival observed in the two groups. Overall, these results indicate that the expression of ZAP-70 alters the homing of malignant B-cells mainly by inducing a massive infiltration of the bone marrow.

**Table 1:** Percentage of GFP-positive cells in the organs from mice injected with Raji GFP or Raji GFP-ZAP-70 cells as assessed by flow cytometry.

	Raji GFP (%)	Raji GFP-ZAP-70 (%)	Fold change	*P*
**Bone marrow**	2.91±1.49	67.07±5.7	23.1	<0.001
**Brain**	0.03±0.017	0.19±0.05	5.4	0.006
**Spleen**	0.02±0.003	0.09±0.017	5.2	<0.001
**Lung**	0.10±0.046	0.24±0.079	2.37	0.007
**Liver**	0.02±0.006	0.05±0.012	1.9	0.028
**Kidney**	0.01±0.018	0.06±0.03	4.17	0.095
**Submaxilar LN**	0.02±0.045	0.05±0.02	3.29	0.069
**Blood**	0.58±0.26	1.03±0.46	1.15	0.095

Results are shown as mean ± SEM. LN: lymph node.

### *In vitro* migration toward CXCL12 and to BMSC is increased in B-cells expressing ZAP-70

**Figure 3:**
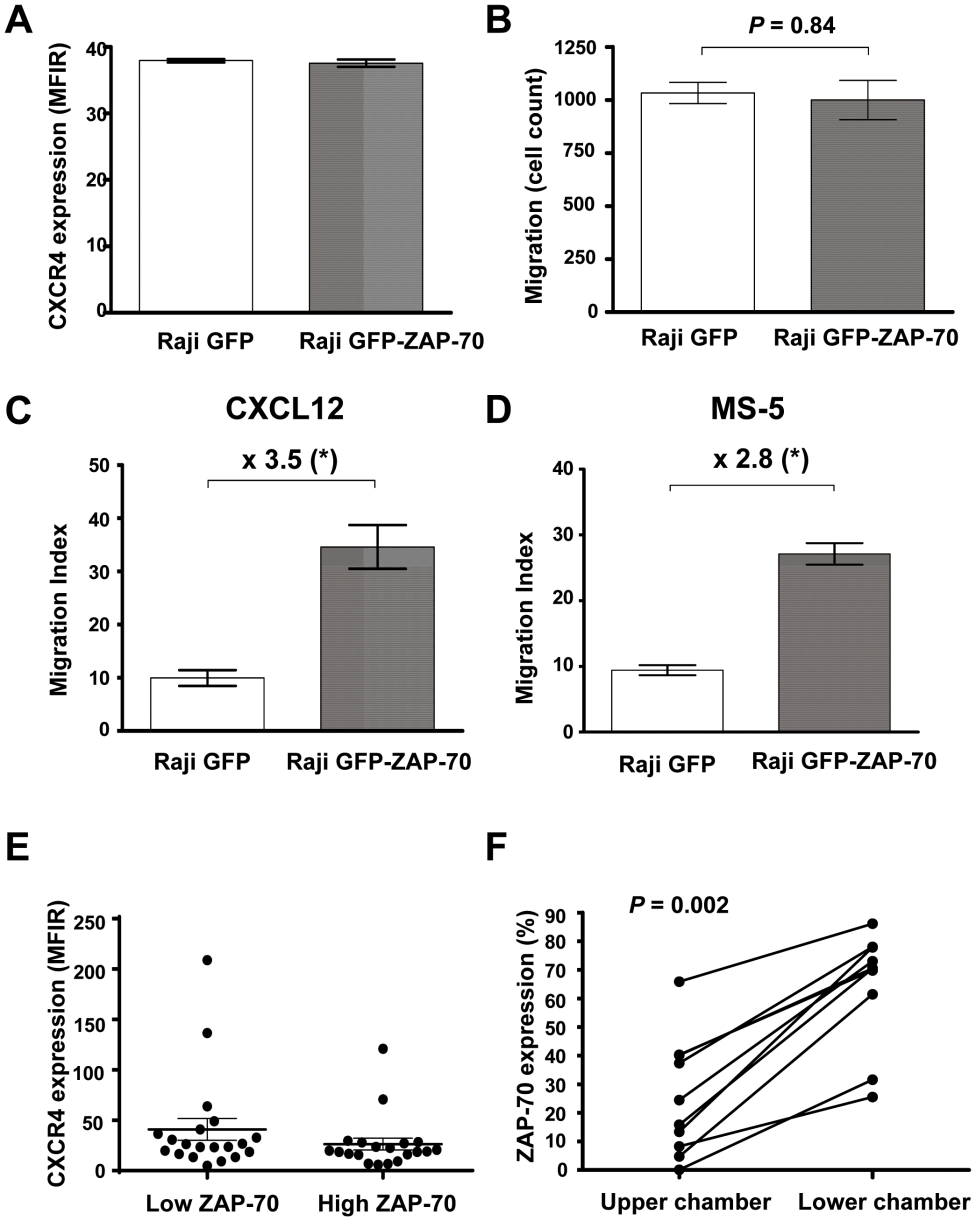
ZAP-70-positive cells have increased migrative capacity toward CXCL12 and the BMSC cell line MS-5. (A) Mean fluorescence intensity ratio (MFIR) of CXCR4 surface staining in Raji GFP and Raji GFP-ZAP-70. (B) Raji transfectants were subjected to migration assay for 4 hours at 37°C in 5% CO_2_ with medium containing CXCL12 (100 ng/mL) in the upper and lower chamber. Migrated cells were counted with a Navios cytometer under a defined flow rate for 5 minutes. (C).Raji transfectants were subjected to migration assay toward CXCL12 (100 ng/mL) or the stromal cell line MS-5 (D) for 4 hours at 37°C in 5% CO_2_ and cells in the lower chamber were counted with a Navios cytometer under a defined flow rate for 5 minutes. *P<.05 (Mann-Whitney test). Results are shown as the mean ± SEM of at least 4 independent experiments. (E) MFIR of CXCR4 surface staining in 20 patients with low ZAP-70 expression and 20 patients with high ZAP-70 expression. Results in the graph show mean ± SEM. (F) Peripheral blood mononuclear cells from 10 patients with CLL were subjected to migration assays toward CXCL12 (100 ng/mL) for 6 hours at 37°C in 5% CO_2_. The percentage of CD19+/CD5+/CD3- CLL cells expressing ZAP-70 was determined in the cellular fraction remaining in the upper chamber and in the cellular fraction of transmigrated cells for each patient by flow cytometry. Wilcoxon test was used, and *P*<.05 was considered significant.

The main attracting chemokine regulating trafficking of lymphocytes to the bone marrow is CXCL12, also known as stromal-derived factor-1α (SDF-1α) [26], [27]. In order to further study the higher bone marrow infiltration found in mice injected with Raji B-cells expressing ZAP-70, we analyzed their expression of CXCR4, the receptor for CXCL12, and their capacity to migrate *in vitro* toward both CXCL12 and the BMSC cell line MS-5. As we had previously observed [16], ZAP-70 ectopic expression did not influence the surface expression of CXCR4 protein (Figure 3A). Subsequently, we analyzed the capacity to migrate toward 100 ng/mL CXCL12 performing transmigration assays across bare polycarbonate membranes. Firstly, in order to rule out a potential chemokinetic effect caused by ZAP-70, we performed a transmigration assay adding CXCL12 on both sides of the membrane. We observed that in the absence of a CXCL12 concentration gradient, there was no difference in the basal rate of transmigration across membranes between the two cell lines (Figure 3B). In contrast, when CXCL12 was present only in the bottom well (Figure 3C) Raji cells expressing ZAP-70 had a migration index 3.5 times higher than Raji cells without ZAP-70 (migration index: 34.6±4.1 vs. 9.9±1.5; *P* = .0079). In addition, the migration index toward the BMSC line MS-5 was 2.8 fold higher in Raji GFP-ZAP-70 cells compared to Raji GFP cells (migration index: 27.1±1.6 vs. 9.4±0.7; *P* = .028; Figure 3D). These results demonstrate that the expression of ZAP-70 enhances the migration of Raji B-cells toward both CXCL12 and BMSCs, which is in line with the increased infiltration of the bone marrow observed in mice inoculated with Raji GFP-ZAP-70 cells.

### Subclones of primary CLL cells expressing ZAP-70 have enhanced migrative capacity toward CXCL12

In order to further study the role of ZAP-70 in B-cell migration toward CXCL12, we analyzed a series of primary cells from patients with CLL. Cells from patients with CLL and high expression of ZAP-70 are reported to have enhanced migration toward CXCL12 upon BCR stimulation [14] and increased signaling and survival upon CXCR4 stimulation compared to cells from patients with low ZAP-70 [12], [15]. Despite this, no significant differences in CXCR4 expression have been found between groups of patients with high or low ZAP-70 expression [12], [15]. Accordingly, in a larger series comparing CXCR4 expression between patients with high (≥20%; n = 20) and low (n = 20) expression of ZAP-70, we did not find any significant difference as well (Figure 3E). Therefore, we investigated whether the enhanced migrative capacity was related to the differential migration of subclones with higher expression of ZAP-70. For this, chemotaxis assay toward CXCL12 using CLL cells from 10 patients was performed, and the expression of ZAP-70 in the transmigrated CLL cells, defined as CD19+/CD5+/CD3−, was assessed by flow cytometry. In agreement with what we observed in Raji cells, in all cases the percentage of ZAP-70-positive cells was significantly higher in cells that had migrated compared to the unresponsive cells remaining in the upper chamber (Figure 3F). These results indicate that, despite that CXCR4 expression is similar between high and low ZAP-70 expressing CLL cells, ZAP-70-positive CLL cells have an enhanced ability to respond to and to migrate toward CXCL12.

### CXCR4 signaling is enhanced in B-cells expressing ZAP-70

**Figure 4:**
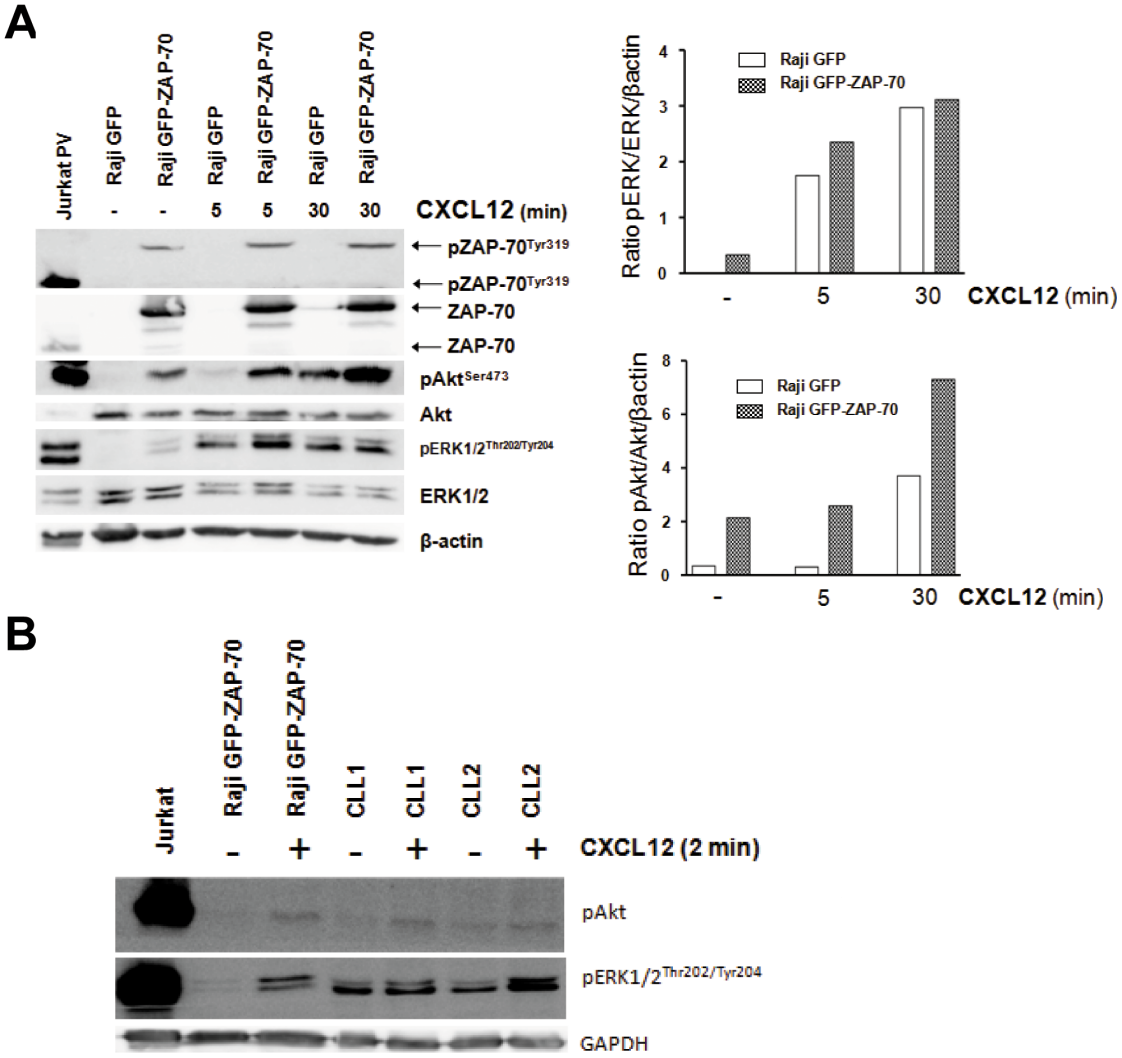
CXCR4 signaling is enhanced in B-cells expressing ZAP-70. (A) Raji transfectants were stimulated with 100 ng/mL of CXCL12 for 5 or 30 minutes and protein phosphorylation was analyzed by western blotting. Enhanced phosphorylation of ZAP-70, Akt and ERK1/2 was observed in the Raji GFP-ZAP-70 cells stimulated with CXCL12. The figure shows a representative example of 3 independent experiments. (B) Raji transfectants and primary CLL cells were stimulated with 100 ng/mL of CXCL12 for 2 minutes and protein phosphorylation was analyzed by western blotting. Similar activation of Akt and ERK1/2 proteins was observed between Raji and primary CLL cells. Jurkat cells treated with pervanadate (PV) were used as positive controls. Note that ZAP-70-GFP fusion protein expressed by Raji GFP-ZAP-70 cells is 97 KDa.

We observed that Raji cells and primary CLL cells expressing ZAP-70 protein showed an enhanced migration toward CXCL12, despite having comparable levels of CXCR4 expression, indicating that differences must be because of ZAP-70 influence in downstream signaling after CXCR4 stimulation. In fact, CXCR4 engagement induces enhanced signaling in CLL cells from patients with high ZAP-70 [12], [15]. CXCR4 stimulation in malignant B-cells activates different signaling pathways, such as the MAPK and PI3K-Akt pathways, which, in addition to migration, can lead to cell growth and survival [12], [15], [28], [29]. In order to specifically analyze the involvement of ZAP-70 in CXCR4 signaling, we stimulated Raji GFP and Raji GFP-ZAP-70 cells with 100 ng/mL of CXCL12 and observed an increased phosphorylation of ZAP-70 at activating residue Tyr319 in Raji GFP-ZAP-70, which translated into an enhanced activation of ERK1/2 and Akt proteins compared to Raji GFP (Figure 4A). ERK1/2 protein phosphorylation was increased after 5 minutes of activation, whereas after 30 minutes the level of activation was similar between Raji GFP and Raji GFP-ZAP-70 cells. As for Akt protein, the increased phosphorylation was evident at both 5 and 30 minutes. These results show that ZAP-70 protein can actively participate in the CXCR4 signaling pathway and that it enhances the activation of proteins from the MAPK and PI3K-Akt pathways; this phenomenon may be responsible for the increased response to CXCR4 stimulation in B-cells expressing ZAP-70 in terms of migrative capacity toward the bone marrow observed *in vivo* and toward CXCL12 and BMSCs observed *in vitro*. In addition, the activation status of both MAPK and PI3K pathways after CXCR4 stimulation was compared in Raji and primary CLL cells with high expression of ZAP-70. After stimulation with 100 ng/mL CXCL12 for 2 minutes, similar levels of activation of ERK1/2 and Akt proteins were found (Figure 4B), indicating that it is unlikely that the phenotype observed in Raji cells is due to supra-physiological stimulation of the pathway after ectopic ZAP-70 expression.

### Effect of CXCR4 stimulation in the proliferation of Raji transfectants

**Figure 5:**
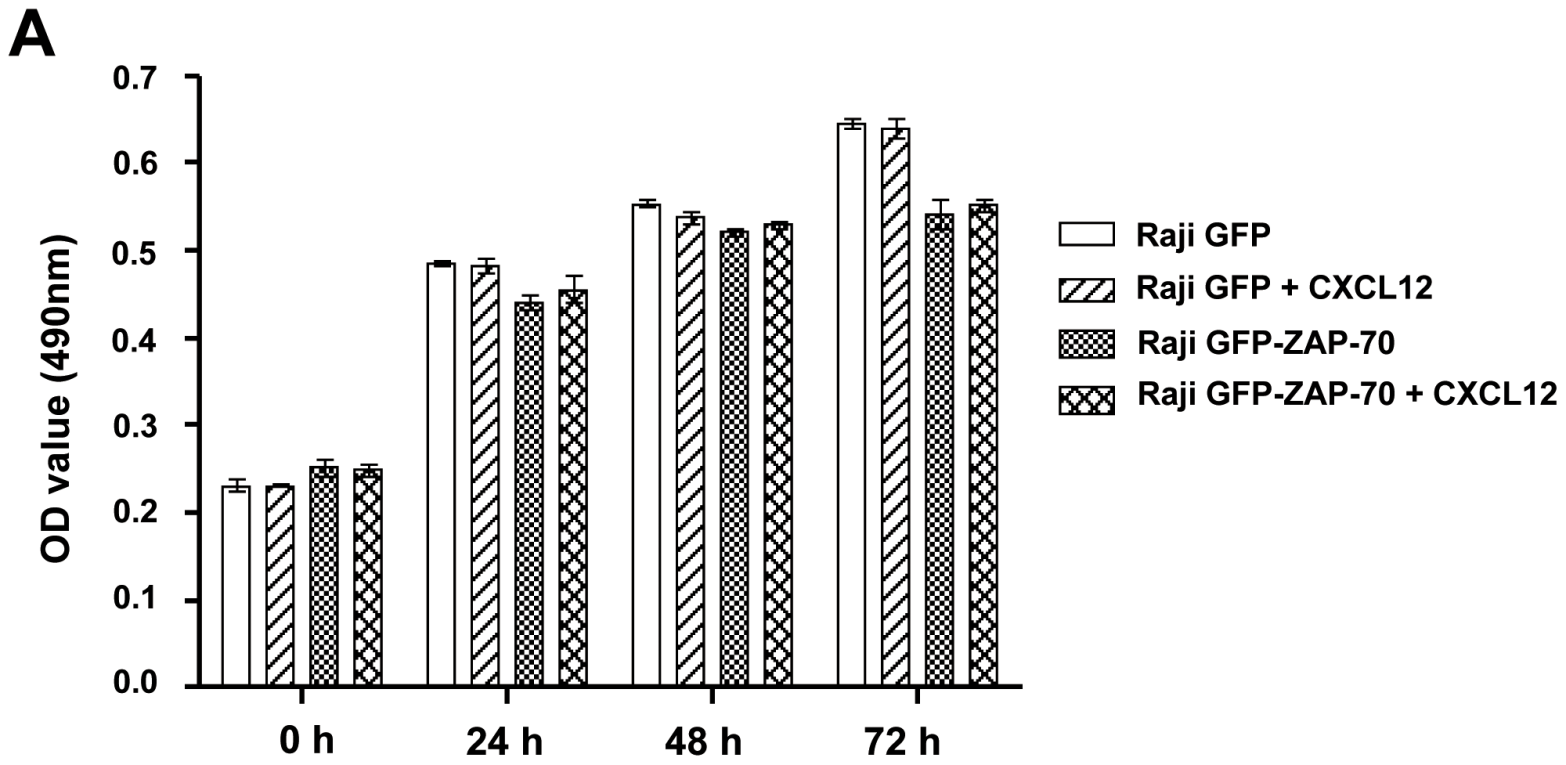
CXCL12 does not affect proliferation of Raji cells. Proliferation of Raji cells exposed to chemokine CXCL12 after 24, 48 and 72-assay. The mean optical density (OD) values at 490 nm ± SEM from 5 replicates are depicted in the graph.

We observed an increased infiltration of the bone marrow in animals injected with ZAP-70-positive malignant B-cells, which was related to an enhanced capacity to respond to CXCR4 stimulation in terms of migration and signaling. In addition, an increased response in terms of proliferation after exposure to CXCL12 in the bone marrow could also contribute to the observed phenotype. In order to test this hypothesis, we compared the *in vitro* proliferation of Raji GFP and Raji GFP-ZAP-70 cells after 24, 48 and 72 hours of exposition to CXCL12. As depicted in Figure 5, CXCL12 did not affect the proliferation of Raji cells, regardless of ZAP-70 expression indicating that enhanced migration but not proliferation is probably the main contributor to the increased infiltration of the bone marrow observed in mice injected with Raji GFP-ZAP-70 cells.

### CXCR4 blockage drastically reduces *in vitro* migration of malignant B-cells toward CXCL12 and to BMSC

**Figure 6:**
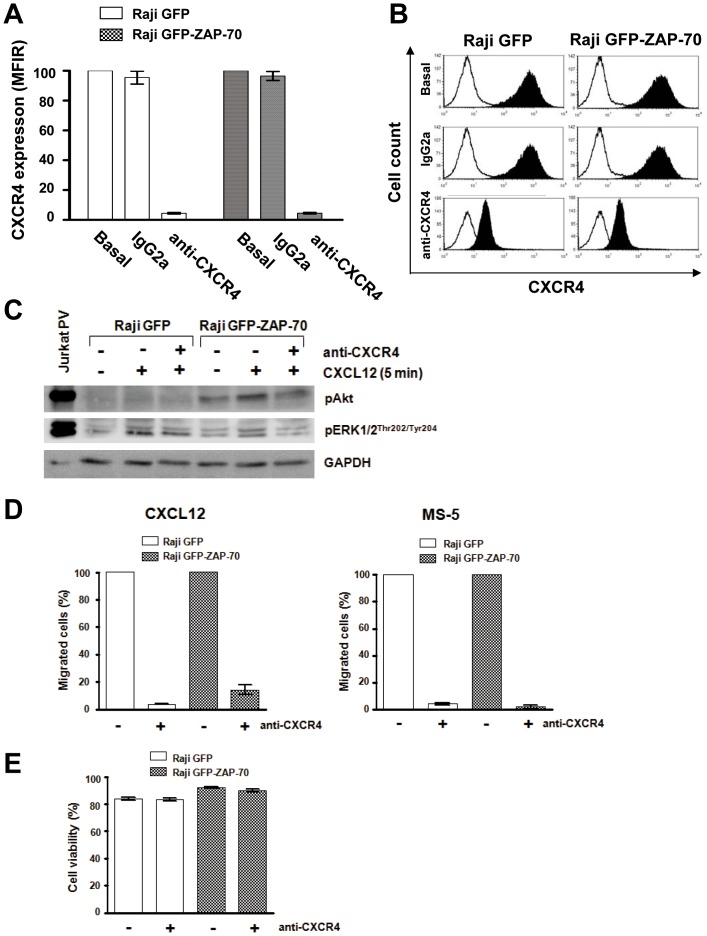
Anti-CXCR4 antibody reduces CXCR4 surface expression, and blocks migration toward CXCL12 and BMSCs. (A) Relative MFIR of CXCR4 surface staining after incubation of Raji transfectants with 100 µg/mL of anti-CXCR4 antibody for 30 minutes. (B) Flow cytometry histograms showing the reduction in CXCR4 expression after incubation with neutralizing anti-CXCR4 antibody. White histograms represent negative unstained controls. (C) CXCR4-blocked cells were stimulated with 100 ng/mL of CXCL12 for 5 minutes and activation of Akt and ERK1/2 proteins analyzed by western blotting. (D) CXCR4-blocked cells were subjected to migration assay for 4 hours at 37°C in 5% CO_2_ toward 100 ng/mL of CXCL12 (left panel) or the stromal MS-5 cell line (right panel). Cells in the lower chamber were counted by flow cytometry under a defined flow rate for 5 minutes. Number of migrated cells treated with isotypic control was considered 100%. Results are shown as the mean ± SEM of 4 independent experiments. (E) Cell viability was measured by Annexin V-PI staining in cells incubated with 100 µg/mL of anti-CXCR4 or isotypic control for 48 hours. Results are shown as the mean ± SEM of 4 independent experiments

To confirm that the increased response to CXCL12 and to BMSCs observed in Raji B-cells expressing ZAP-70 is specifically mediated by CXCR4, we blocked its surface expression with a monoclonal antibody [30]. As depicted in Figures 6A and 6B, a relative reduction of surface expression superior to 95% in both cell lines after treatment with anti-CXCR4 was observed. Moreover, the phosphorylation of Akt and ERK1/2 proteins after stimulation of CXCR4 with CXCL12 was also impaired after treatment with anti-CXCR4 regardless of ZAP-70 expression (Figure 6C). Blocking of CXCR4 caused a severe drop in the number of migrated cells toward CXCL12 (Figure 6D, left panel) in both cell lines Raji GFP and Raji GFP-ZAP-70 (96% and 86% mean reduction, respectively). Similarly, migration toward BMSC MS-5 was also strongly impaired with a mean reduction of 95% for Raji GFP and 98% for Raji GFP-ZAP-70 (Figure 6D, right panel). Importantly, the inhibition of CXCR4 did not affect the survival of the cells (Figure 6E). These results indicate that the CXCL12/CXCR4 axis plays a crucial role in the migration of malignant Raji B-cells toward a bone marrow-like microenvironment.

## Discussion

Accumulating evidence demonstrates that the interaction of CLL cells with the microenvironment plays a relevant role in the natural history of the disease by promoting survival, proliferation, and resistance to chemotherapy of the malignant cells [6]–[8]. Importantly, we and others have reported that ZAP-70 protein, highly expressed in patients with adverse prognosis, is participating in this crosstalk by enhancing several signals from the microenvironment, such as signaling from the BCR and from diverse chemokines [12]–[17]. However, the comparison of patients with high vs. low ZAP-70 can mask the actual role of ZAP-70 protein, since many other biological factors can be involved. In addition, the methodology used to define high and low ZAP-70 varies among laboratories. Therefore, with the aim of studying the specific contribution of ZAP-70 protein to the crosstalk between malignant B-cells and the microenvironment, we stably transfected malignant B-cells with ZAP-70 protein and studied the phenotypic effects. Since the establishment of a CLL cell line is still an unsolved issue (reviewed in ref [31]) we used a Burkitt's lymphoma cell line as a model of malignant mature B-lymphocyte, as previously done in several reports [16], [32]. Using this model, we previously described the direct role of ZAP-70 protein in enhancing response to the BCR and to CCR7 stimulation [16]. Herein, we studied the role of ZAP-70 in the *in vivo* homing of B-cells in a xenograft model by intravenously inoculating Raji B-cells expressing ZAP-70 into SCID mice. Firstly, we observed that ZAP-70 expression did not influence mice survival since the capacity of Raji cells to infiltrate the central nervous system, which is the main cause of paralysis and death in this mice model, was not altered. By contrast, the capacity of Raji B-cells to infiltrate the bone marrow was greatly enhanced by the expression of ZAP-70. The striking difference prompted us to further study the role of ZAP-70 in the response of malignant B-cells to CXCL12, the main attracting chemokine secreted by stromal cells in the bone marrow [26], [27].

As previously described [12], [15] in our B-cell system and in primary CLL cells, we observed similar levels of CXCR4 regardless ZAP-70 expression. In spite of that, the capacity of Raji B-cells to migrate toward CXCL12 and to BMSCs *in vitro* was highly enhanced by the expression of ZAP-70. Moreover, despite the similar CXCR4 levels, the analysis of migration toward CXCL12 of primary CLL cells revealed that those CLL cells subclones expressing ZAP-70 where enriched among cells with migrative capacity, highlighting the role of ZAP-70 in CXCR4-mediated migration also in primary CLL cells. Variable results have been reported about the relationship between ZAP-70 and CXCL12-induced migration in primary CLL samples. In this sense, ZAP-70-positive cells have been also found to be enriched within the fraction of primary CLL cells that migrate towards mesenquimal stromal cells conditioned media [17]; however, CLL cells from patients with high ZAP-70 expression only showed significantly higher migration toward CXCL12 after BCR stimulation [12], [14], [17]. Since CXCR4 expression seems not to be influenced by ZAP-70, we hypothesized that the differences observed in the migrative capacity both *in vivo* and *in vitro* might be due to increased signaling upon CXCR4 stimulation in cells expressing ZAP-70. In this sense, we observed that, in Raji B-cells, CXCL12 induced a rapid activation of ZAP-70 and, subsequently, downstream signaling elements such as Akt and ERK1/2 proteins showed enhanced activation as well. Although ZAP-70 protein was initially described to specifically participate in T lymphocytes and NK cells signaling [33], it was later found to also participate in the BCR signaling in CLL cells [32], [34], [35]. In addition, several reports have already shown that ZAP-70 can directly participate in CXCR4 signaling in T lymphocytes [36], [37]. Interestingly, other tyrosine kinases participating in the BCR signaling pathway in CLL, such as Syk, BTK, and PI3K have also been implicated in CXCR4 signaling and migration toward CXCL12 in primary CLL cells *in vitro* [38]–[42]. This is of special interest since the inhibition of BCR-related kinases that also participate in the CXCR4 signaling is showing promising results in preliminary clinical trials in patients with CLL [43]–[45]. Of note, mobilization of CLL cells into peripheral blood is observed in patients treated with such inhibitors, where an initial lymphocytosis is observed, thus further supporting *in vivo* the involvement of these proteins in the CXCR4 pathway. In our experiments, when we neutralized CXCR4 in Raji B-cells with a monoclonal antibody, *in vitro* migration toward both CXCL12 and the BMSC line was almost completely impaired regardless ZAP-70 expression, highlighting the importance of the CXCL12/CXCR4 axis in driving the migration of B-lymphocytes to the bone marrow.

In summary, this study demonstrates that ZAP-70 protein is responsible for an enhanced cellular migration into the bone marrow caused by an amplified response to CXCR4 stimulation as regards signaling and migration in this cellular model. In patients with CLL, this mechanism would facilitate the access of malignant cells to receive additional survival, anti-apoptotic and proliferative signals that can be found in the supportive microenvironment of the bone marrow. Of note, a correlation between higher bone marrow infiltration and high expression of ZAP-70 has been observed in patients with CLL [46]. Further elucidation of the role and modulation of signals from the microenvironment and especially from CXCR4 can contribute to enlighten the biology behind the adverse clinical outcome of patients with CLL and high expression of ZAP-70, and can potentially be exploited for the development of new treatments.
